# Prognostic Value of Metabolic Tumor Volume and Heterogeneity Index in Diffuse Large B-Cell Lymphoma

**DOI:** 10.3390/medicina61081370

**Published:** 2025-07-29

**Authors:** Ali Alper Solmaz, Ilhan Birsenogul, Aygul Polat Kelle, Pinar Peker, Burcu Arslan Benli, Serdar Ata, Mahmut Bakir Koyuncu, Mustafa Gurbuz, Ali Ogul, Berna Bozkurt Duman, Timucin Cil

**Affiliations:** 1 Medical Oncology Department, Adana City Hospital, Adana 01370, Turkey; pnnarrrr@gmail.com (P.P.); brcarsln51@gmail.com (B.A.B.); drserdarata@gmail.com (S.A.); berboz@hotmail.com (B.B.D.); timucin.cil@sbu.edu.tr (T.C.); 2 Internal Medicine Department, Adana City Hospital, Adana 01370, Turkey; ilhanbirsenogul@gmail.com; 3 Nuclear Medicine Department, Adana City Hospital, Adana 01370, Turkey; dolunay_75@hotmail.com; 4 Health Services Vocational School, Toros University, Mersin 33140, Turkey; mahmutbakirkoyuncu@gmail.com; 5 Medical Oncology Department, Faculty of Medicine, Baskent University, Adana 01120, Turkey; mustafagurbuz123@hotmail.com; 6 Medical Oncology Department, Faculty of Medicine, Mersin University, Mersin 33110, Turkey; mdaliogul@gmail.com

**Keywords:** non-hodgkin lymphoma, metabolic tumor volume, heterogeneity index, prognostic factors, PET/CT

## Abstract

*Background and Objectives*: Metabolic tumor volume (MTV) and inflammation-based indices have recently gained attention as potential prognostic markers of diffuse large B-cell lymphoma (DLBCL). We aimed to evaluate the prognostic significance of metabolic and systemic inflammatory parameters in predicting treatment response, relapse, and overall survival (OS) in patients with DLBCL. *Materials and Methods*: This retrospective cohort study included 70 patients with DLBCL. Clinical characteristics, laboratory values, and metabolic parameters, including maximum standardized uptake value (SUVmax^liver^ and SUVmax), heterogeneity indices HI1 and HI2, and MTV were analyzed. Survival outcomes were assessed using Kaplan–Meier and log-rank tests. Receiver operating characteristic analyses helped evaluate the diagnostic performance of the selected biomarkers in predicting relapse and mortality. Univariate and multivariate logistic regression analyses were conducted to identify the independent predictors. *Results*: The mean OS and mean relapse-free survival (RFS) were 71.6 ± 7.4 and 38.7 ± 2.9 months, respectively. SUVmax^liver^ ≤ 22 and HI2 > 62.3 were associated with a significantly shorter OS. High lactate dehydrogenase (LDH) levels and HI2 > 87.9 were significantly associated with a reduced RFS. LDH, SUVmax^liver^, and HI2 had a significant predictive value for relapse. SUVmax^liver^ and HI2 levels were also predictive of mortality; SUVmax^liver^ ≤ 22 and HI2 > 62.3 independently predicted mortality, while HI2 > 87.9 independently predicted relapse. MTV was not significantly associated with survival. *Conclusions*: Metabolic tumor burden and inflammation-based markers, particularly SUVmax^liver^ and HI2, are significant prognostic indicators of DLBCL and may enhance risk stratification and aid in identifying patients with an increased risk of relapse or mortality, potentially guiding personalized therapy.

## 1. Introduction

Malignant lymphomas are caused by the clonal proliferation of lymphocytes. Diffuse large B-cell lymphoma (DLBCL) is a heterogeneous group of lymphoid malignancies originating from B cells. The clinical course of DLBCL varies widely, ranging from subtypes responding well to treatment with prolonged survival to aggressive subtypes associated with rapid disease progression and poor outcomes. Given this heterogeneity, the identification of reliable prognostic biomarkers is crucial for risk stratification and treatment optimization [1].

The International Prognostic Index (IPI) has been widely used as a clinical tool to predict the outcomes of DLBCL, incorporating factors such as age, disease stage, serum lactate dehydrogenase (LDH) level, performance status, and extranodal involvement. Although the IPI remains the cornerstone of DLBCL prognosis, its predictive power is limited, particularly in the era of advanced molecular and imaging techniques. Traditional risk stratification methods do not fully capture tumor biology, metabolic activity, or microenvironmental heterogeneity, leading to the need for more objective and dynamic prognostic tools [2].

Positron emission tomography/computed tomography (PET/CT) using 18F-fluorodeoxyglucose (18F-FDG) has emerged as a standard imaging modality for staging, response assessment, and prognostication of DLBCL. Qualitative evaluation of PET scans requires attention to the details in the thresholding of images [3,4]. Unlike conventional anatomical imaging modalities, PET/CT provides functional and metabolic insights into tumor behavior, reflecting tumor burden, proliferation, and treatment response. Several PET-derived parameters, including maximum standardized uptake value (SUVmax), metabolic tumor volume (MTV), and total lesion glycolysis (TLG), have been explored as potential prognostic markers. FDG parameters, such as tumor volume/metabolism, TLG, SUVmax, and MTV, have been widely investigated. TLG represents the product of the mean SUV and MTV, while MTV represents the extent of active uptake of 18F-FDG by tumor tissue [5,6]. Among these, MTV has gained particular attention because of its ability to quantify the volumetric extent of metabolically active tumor tissues, making MTV measurement a superior predictor of outcomes compared to single-point SUVmax measurements [7,8].

Although traditional parameters can reflect cancer heterogeneity characteristics to some extent, they have certain limitations. SUV can reflect activity at only one point within the tumor rather than the overall metabolism of the tumor. MTV and TLG can compensate for this shortcoming and can reflect metabolic information in the entire tumor, which may be more accurate than single-voxel measurements for tumor characterization [9].

MTV reflects not only the tumor burden but also the metabolic activity of the entire lesion, which is associated with disease aggressiveness and treatment response. Previous studies have shown that a higher MTV is associated with a shorter progression-free survival (PFS) and overall survival (OS) in DLBCL. However, the MTV threshold remains unclear, and further studies are needed to improve its prognostic utility. In Japanese patients with R-CHOP-treated DLBCL, Nakaya et al. concluded that this index did not reflect PFS in their cohort [10].

Beyond the MTV, tumor heterogeneity has emerged as another critical factor that influences cancer progression and treatment resistance. The heterogeneity index (HI), derived from PET/CT imaging, quantifies variations in metabolic activity within the tumor and provides insights into the tumor microenvironment complexity. Increased metabolic heterogeneity is often indicative of intratumoral diversity, including regions of necrosis, hypoxia, differential cellular proliferation, and varying glucose uptake patterns. High HI values have been linked to aggressive tumor phenotypes, treatment resistance, and poor prognosis in several malignancies, including lung and breast cancers. However, the prognostic significance of HI for DLBCL remains unclear [11,12].

This study aimed to investigate the prognostic significance of MTV and HI in patients with DLBCL and to assess their impact on OS and relapse-free survival (RFS). We hypothesized that high MTV and metabolic heterogeneity are associated with poor clinical outcomes. By integrating PET/CT-derived biomarkers into prognostic models, we sought to enhance risk stratification, refine treatment strategies, and improve outcomes in patients with DLBCL.

## 2. Materials and Methods

This retrospective study included 70 patients diagnosed with DLBCL who were followed at a single tertiary care center. Patients were eligible if they had histopathologically confirmed DLBCL and had undergone baseline 18F-FDG PET/CT imaging before chemotherapy initiation. All patients received standard first-line treatment, primarily R-CHOP or R-EPOCH regimens, based on clinical presentation and the physician’s discretion. Demographic data, clinical stage, Eastern Cooperative Oncology Group (ECOG) performance status, treatment regimens, response to therapy, relapse, and mortality data were extracted from electronic medical records. Patients with insufficient imaging or clinical data were excluded.

The clinical variables recorded were age, sex, disease stage according to the Ann Arbor classification, and bone marrow involvement. Laboratory values at diagnosis, such as serum LDH and albumin levels, white blood cell (WBC) count, absolute neutrophil count, lymphocyte count, platelet count, and Ki-67 proliferation index, were collected for each patient. Based on these values, several inflammation-based indices, including the neutrophil-to-lymphocyte ratio (NLR), derived NLR (dNLR), systemic immune inflammation index (SII), and prognostic nutritional index (PNI), were calculated.

### 2.1. PET/CT Technique

Imaging was performed using a PET/CT scanner (Biography, Ingenuity TF Philips, Cleveland, OH, USA) at the Nuclear Medicine Department of Adana City Hospital. Before the scan, patients were instructed to fast for a minimum of 6 h. Diuretics were not administered during the preparation period. Patients with blood glucose levels < 200 mg/dL received an intravenous injection of approximately 3.7 MBq/kg (range: 222–444 MBq) of 18F-FDG. One hour after the injection, supine PET/CT imaging was performed. CT scans were obtained without intravenous contrast and were used solely for attenuation correction and anatomical reference. The scan range was extended from the skull base to the upper thighs, using the following parameters: 80 mA, 120 kV, and slice thickness of 4 mm. PET acquisition followed, covering 8–10 bed positions with an acquisition time of 2–3 min per bed position. The images were reconstructed using an iterative technique, processed using the Philips e-Soft platform, and developed for PET/CT image analysis. The reconstructed outputs included 3D whole-body maximum intensity projection and cross-sectional images in the coronal, sagittal, and axial planes. Both visual and semiquantitative evaluations were performed by an experienced nuclear medicine expert with 12 years of expertise.

### 2.2. PET/CT Image Analysis

**Figure 1:**
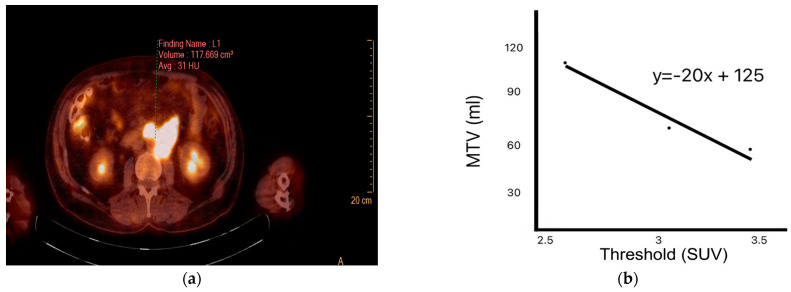
Methods for calculating indices of intratumoral heterogeneity. (**a**) Positron emission tomography (PET) image showing manually drawn metabolic tumor volume (MTV); (**b**) negative slope of the linear regression line obtained from MTV data across several standardized uptake value (SUV) thresholds.

Target lesions were analyzed by defining regions of interest (ROIs) to calculate SUVmax, SUVmean, SUVpeak, and MTV. SUVmax denotes the highest uptake value within the ROI. SUVmean refers to the average uptake across the entire selected region. Using a volumetric method, SUVpeak was calculated based on the voxel with the highest activity. HI1 and HI2 levels were also computed. HI1 represents the coefficient of variation calculated by dividing the standard deviation of the SUV by the SUVmean. HI2 is defined as the negative slope of the linear regression line derived from MTV measurements across varying SUV thresholds (2.5, 3.0, and 3.5) [13] based on a refined approach adapted from previously established methods [14,15]. SUVmax^liver^ and SUVmean^liver^ were calculated by placing an ROI over normal liver tissue on the PET images. SUVmax^liver^ represents the SUVmax within this ROI, whereas SUVmean^liver^ represents the average SUV within the same region (Figure 1) [16].

All clinical, laboratory, and imaging parameters were evaluated in relation to treatment response, relapse status, and OS outcomes. Patients were followed up longitudinally from the time of diagnosis to either the last follow-up or death.

### 2.3. Statistical Analysis

The SPSS software (version 25.0) was used for statistical analysis of the data. Categorical measurements were summarized as counts and percentages, while continuous measurements were summarized as mean and standard deviation (with median and minimum-maximum, 25–75th percentiles where necessary). The Kolmogorov–Smirnov test was used to determine whether the parameters included in the study showed a normal distribution. For parameters that did not show a normal distribution, the Mann–Whitney U test was used. Chi-square and Fisher’s exact tests were used to compare categorical variables. Kaplan–Meier and log-rank tests were used to determine the patient survival rates. The sensitivity and specificity of the relevant parameters were calculated based on the mortality and recurrence variables of the patients included in the study, and the cutoff value was determined by examining the area under the receiver operating characteristic (ROC) curve. To examine the factors affecting mortality and recurrence, logistic regression tests were used in the univariate analysis, and multivariate logistic regression tests were used in the multivariate analysis. For all tests, the statistical significance level was set at *p* < 0.05.

## 3. Results

### 3.1. Baseline Patient Characteristics

This retrospective study included 70 patients diagnosed with DLBCL. The median age at diagnosis was 62 years (range: 26–89 years), and 54.3% of the patients were aged > 60 years. Male patients constituted 58.6% of the cohort. At the time of diagnosis, stage IV disease was the most prevalent, observed in 50% of cases. Most patients (82.9%) received R-CHOP as the first-line chemotherapy regimen, and only a minority (17.1%) received R-EPOCH. Regarding performance status, 52.9% of the patients had an ECOG score of 2 or above. A positive treatment response was achieved in 97.1% of patients, and 48.6% exhibited a complete or near-complete response. During the follow-up period, 30.0% of the patients died and 41.4% experienced disease relapse. Table 1 shows the demographic characteristics of patients.

### 3.2. OS and RFS

The mean OS for the entire cohort was calculated as 71.6 ± 7.4 months (95% CI: 57.2–86.1). The mean RFS was 38.7 ± 2.9 months (95% CI: 32.9–44.6). When subgroup analyses were performed, a significantly lower OS was observed in patients with an SUVmax^liver^ value ≤ 22 (*p* = 0.003) and in those with an HI2 score > 62.3 (*p* = 0.013). LDH levels > 302 IU/L were associated with a significantly shorter RFS (*p* = 0.002). Similarly, patients with HI2 values > 87.9 (*p* = 0.001) experienced a significantly reduced RFS.

ROC curve analysis was conducted to evaluate the diagnostic accuracy of various inflammatory and metabolic parameters for predicting disease relapse. The LDH, SUVmax^liver^, and HI2 levels had statistically significant predictive values (*p* < 0.05). Among these, LDH showed the highest area under the curve (AUC; 0.675), followed by HI2 (0.655) and SUVmax^liver^ (0.647). Notably, an HI2 cutoff value of > 87.9 was associated with the highest sensitivity (79.3%). These findings suggest that these markers, especially LDH and HI2, could be helpful for the early identification of patients at an increased risk of relapse.

### 3.3. Predictive Role of ROC-Based Biomarkers in Mortality

In mortality prediction, SUVmax^liver^ and HI2 also demonstrated significant diagnostic performance. SUVmax^liver^ ≤ 22 yielded the highest AUC (0.726), indicating strong predictive power, followed by HI2 > 62.3 (AUC = 0.661). SUVmax^liver^ ≤ 22 had the highest sensitivity (85.7%), whereas HI2 > 62.3 showed the highest specificity (85.7%). These results underscore the utility of SUVmax^liver^ and HI2 as prognostic indicators of survival in patients with DLBCL.

### 3.4. Associations with Treatment Response

Patients who achieved a complete or near-complete treatment response at the first interim analysis were characterized by a lower frequency of ECOG ≥ 2 (*p* < 0.001), lower Ki-67 expression levels (*p* = 0.038), and lower SUVmax values (*p* = 0.029). Additionally, the prevalence of Ki-67 expression < 70% was significantly higher in this group (*p* = 0.035). Other clinical or laboratory parameters showed no significant differences between the response groups (*p* > 0.05), suggesting that metabolic and proliferative tumor characteristics may better predict the response than traditional clinical variables.

### 3.5. Factors Associated with Relapse

**Figure 2:**
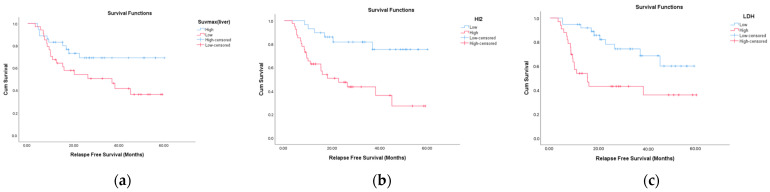
Relapse-free survival (RFS) according to positron emission tomography/computed tomography (PET/CT) parameters and lactate dehydrogenase (LDH). Patients with relapse had significantly higher median LDH levels (*p* = 0.013) and HI2 scores (*p* = 0.028). In contrast, the values of SUVmax^liver^ (*p* = 0.037) were significantly lower in these patients. Kaplan–Meier survival curves showing relapse-free survival differences based on (**a**) SUVmax^liver^ (SUVmax^liver^ ≤ 21 vs. > 21), (**b**) Heterogeneity Index 2 (HI2 ≤ 87.9 vs. > 87.9), and (**c**) LDH (LDH ≤ 301 vs. > 302).

Patients with relapse had a significantly higher mortality rate (72.4%, *p* < 0.001), median LDH levels (*p* = 0.013), and HI2 scores (*p* = 0.028). In contrast, the values of SUVmax^liver^ (*p* = 0.037) were significantly lower in these patients. These findings suggest that elevated systemic inflammation (as reflected by HI2 and LDH levels) and lower metabolic activity in the liver (as reflected by the SUVmax^liver^) were associated with disease recurrence (Figure 2).

### 3.6. Factors Associated with Mortality

**Figure 3:**
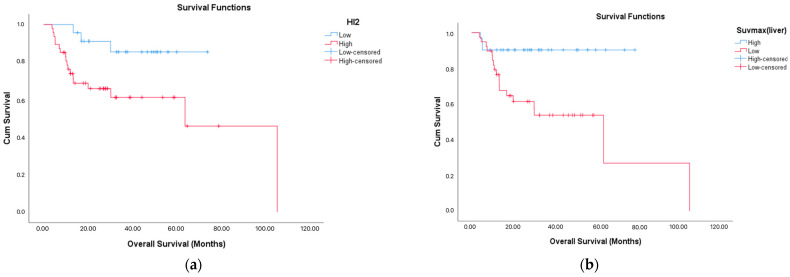
Overall survival (OS) according to PET/CT parameters. Overall survival was shorter in the group with high HI-2 (*p* = 0.016) and in the group with low SUVmaxliver (*p* = 0.003). Kaplan–Meier survival curves showing overall survival differences based on (**a**) HI2 (HI2 ≤ 62.3 vs. > 62.3) and (**b**) SUVmax^liver^ (SUVmax^liver^ ≤ 22 vs. > 22).

Among the 21 patients who died, male sex was significantly more prevalent (*p* = 0.043). These patients were also less likely to have responded to treatment (*p* = 0.028), and all of them experienced disease relapse (*p* < 0.001). Importantly, the SUVmax^liver^ (*p* = 0.003) was significantly lower in this group, suggesting a link between reduced metabolic tumor activity and impaired immune-inflammatory balance with adverse outcomes. No significant differences were observed in other laboratory and clinical variables (Figure 3 and Table 2).

### 3.7. Logistic Regression Analysis

In univariate analysis, SUVmax^liver^ ≤ 22 (OR: 8.000; *p* = 0.002) and HI2 > 62.3 (OR: 0.188; *p* = 0.015) were significantly associated with an increased mortality risk. In the multivariate model, SUVmax^liver^ ≤ 22 (OR: 7.116; *p* = 0.006) and HI2 > 62.3 (OR: 0.225; *p* = 0.043) remained independent predictors (Table 3).

For relapse, univariate analysis identified LDH ≤ 301 (OR: 0.273; *p* = 0.001), SUVmax^liver^ ≤ 21 (OR: 3.293; *p* = 0.019), and HI2 ≤ 87.9 (OR: 0.204; *p* = 0.004) as significant predictors. Multivariate analysis showed that only HI2 ≤ 87.9 was independently associated with relapse (OR, 0.272; *p* = 0.040; Table 4). These regression findings highlight the prognostic utility of tumor heterogeneity and inflammation-based scores (HI2) in predicting relapse and mortality in patients with DLBCL.

**Table 2:** Association Between Mortality and Clinical, Demographic, and Laboratory Characteristics.

Categorical Variables			
Variable	Mortality (+) (n = 21)	Mortality (–) (n = 49)	*p*-Value
Age Group			
>60 years	13 (61.9%)	25 (51.0%)	0.402
≤60 years	8 (38.1%)	24 (49.0%)	
Gender			
Male	16 (76.2%)	25 (51.0%)	**0.043**
Female	5 (23.8%)	24 (49.0%)	
Disease Stage			0.971
Stage 1	4 (19.0%)	10 (20.4%)	
Stage 2	3 (14.3%)	6 (12.2%)	
Stage 3	3 (14.3%)	9 (18.4%)	
Stage 4	11 (52.4%)	24 (49.0%)	
Stages 3–4	14 (66.7%)	33 (67.3%)	0.956
≥1 Comorbidity	6 (28.6%)	17 (34.7%)	0.617
Chemotherapy Regimen			0.678
R-EPOCH	3 (14.3%)	9 (18.4%)	
R-CHOP	18 (85.7%)	40 (81.6%)	
ECOG Performance Score (PS)			0.842
1	9 (42.9%)	24 (49.0%)	
2	6 (28.6%)	14 (28.6%)	
3	6 (28.6%)	11 (22.4%)	
ECOG ≥ 2	12 (57.1%)	25 (51.0%)	0.638
Treatment Response	19 (90.5%)	49 (100.0%)	**0.028**
Complete/Near Complete Response	10 (47.6%)	24 (49.0%)	0.917
Relapse	21 (100.0%)	8 (16.3%)	**<0.001**
Elevated LDH	14 (66.7%)	28 (57.1%)	0.456
IPI Score			0.414
Low–Low Intermediate	15 (71.4%)	30 (61.2%)	
High–High Intermediate	6 (28.6%)	19 (38.8%)	
Bone Marrow Involvement	2 (11.8%)	3 (8.6%)	0.714
Bulky Disease	5 (23.8%)	10 (20.4%)	0.751
**Continuous Variables (Median [25th–75th Percentile])**			
**Variable**	**Mortality (+)**	**Mortality (–)**	***p*** **-Value**
Age (years)	63 (58–67)	60.5 (47–69)	0.453
LDH (U/L)	372 (212–565)	297 (206–432)	0.112
Albumin (g/dL)	3.5 (3.2–3.8)	3.7 (3.2–4.12)	0.230
WBC (/mm^3^)	8800 (6700–9800)	9500 (7775–12,350)	0.290
Neutrophils (/mm^3^)	5800 (4300–7600)	6650 (4775–8525)	0.513
Lymphocytes (/mm^3^)	1300 (600–1700)	1400 (975–2300)	0.186
Platelets (/mm^3^)	276,000 (256 k–365 k)	308,000 (259 k–415.75 k)	0.465
SUVmax^liver^	19 (13–22)	24 (19–27.25)	**0.003**
SUVmax	148 (21–256)	132 (47.5–204)	0.974
SUVpeak	138 (46–236)	137.5 (70–235.25)	0.853
SUVmean	9.6 (7.6–12.7)	6.8 (5.07–10.4)	0.088
SUVmean^liver^	18 (15–19)	21 (17–23.5)	0.292
MTV2.5	1089 (560–2028)	1498.5 (413.75–4649.5)	0.754
MTV3	1378 (644–6633)	1432.5 (242.25–4161.75)	0.097
MTV3.5	1194 (725–5358)	636 (137.5–3628)	0.121
SUV SD	3.4 (1.8–4.9)	2.4 (1.5–4.0)	0.407
PNI	4.8 (3.9–5.1)	5.3 (4–6.43)	0.144
SII	1319 (682–2650)	1434.5 (566.25–2519.5)	0.686
NLR	5.15 (2.63–8.63)	4.38 (1.98–7.98)	0.401
HI1	0.3 (0.24–0.39)	0.31 (0.25–0.38)	0.744
HI2	185.5 (27–476)	93.5 (5–2044)	**0.016**
Ki-67 (%)	75 (50–80)	75 (65–80)	0.475

**Table 3:** Logistic regression analysis of variables affecting mortality.

	Univariate			Multivariate		
	Odd Ratio	95% CI	*p*	Odd Ratio	95% CI	*p*
SUVmax^liver^ (≤22)						
Low	8.000	2.080–30.763	0.002 **	7.116	1.750–28.932	0.006 **
High						
HI2 (≤62.3)						
Low	0.188	0.049–0.723	0.015 *	0.225	0.053–0.955	0.043 *
High						

* *p* < 0.05, ** *p* < 0.01, logistic regression, multivariate logistic regression.

**Table 4:** Logistic regression analysis of variables affecting relapse.

	Univariate			Multivariate		
	Odd Ratio	95% CI	*p*	Odd Ratio	95% CI	*p*
LDH (≤301)						
Low	0.273	0.100–0.743	0.001 **	0.462	0.145–1.467	0.190
High						
SUVmax^liver^ (≤21)						
Low	3.293	1.218–8.908	0.019 *	2.577	0.841–7.899	0.098
High						
HI2 (≤87.9)						
Low	0.204	0.069–0.607	0.004 **	0.272	0.079–0.940	0.040 *
High						

* *p* < 0.05, ** *p* < 0.01, logistic regression, multivariate logistic regression.

## 4. Discussion

In this study, we investigated the prognostic value of metabolic and inflammation-related parameters, including MTV, HI, SUVmax^liver^, and LDH levels, in patients with DLBCL. The findings demonstrate that SUVmax liver and HI2 are strong predictors of both OS and RFS.

SUVmax^liver^ values ≤ 22 were independently associated with increased mortality, while HI2 values > 62.3 were also found to be an independent predictor of poor survival outcomes. Additionally, HI2 values exceeding 87.9 were significantly associated with an increased relapse risk. These results are consistent with those of previous studies emphasizing the prognostic role of volumetric and heterogeneity-based PET/CT parameters across various malignancies.

The strong association between elevated HI2 levels and both OS and RFS highlights the significance of tumor heterogeneity in disease progression. Higher HI2 values may reflect hypoxic or necrotic tumor regions and intratumoral variability in metabolic activity, which are often linked to therapeutic resistance and poor outcomes. Similarly, LDH, as an inflammation-related marker, may also be associated with poor prognosis. Although HI-2, which is derived from MTV, and SUVmax^liver^, a liver-based MTV, were found to predict survival in our study, MTV itself did not demonstrate a direct prognostic value for survival.

ROC analyses further confirmed the diagnostic value of SUVmax^liver^ and HI2 in predicting both relapse and mortality. Multivariate logistic regression identified SUVmax^liver^ ≤ 22 and HI2 > 62.3 as independent predictors of mortality, while HI2 > 87.9 was an independent predictor of relapse. These results highlight the potential of PET/CT-derived and inflammation-based markers as tools for enhanced risk stratification in DLBCL.

SUVmax^liver^ showed the highest SUV in the tumor-free area of the liver. In our study, SUVmax^liver^ was associated with both OS and RFS. However, the prediction of mortality using SUVmax^liver^ remains controversial. Since the liver SUV can reflect systemic metabolic activity, it can be associated with poor prognosis. The stability of liver SUV can increase, especially if corrected for blood glucose levels [16]. The relationship between tumor or liver SUV and prognosis has also been demonstrated in lymphoma and colorectal cancer [17]. To definitively establish that SUVmax-liver is a marker of systemic inflammation and has a prognostic impact on survival, it is necessary to validate this association through additional inflammatory markers. Further studies providing stronger evidence on this subject are warranted.

In addition to tumor volume, metabolic heterogeneity has emerged as a key indicator of tumor biology. The HI quantifies variations in metabolic activity within a tumor and provides insights into tumor aggressiveness [18]. Our study found that HI2 values > 87.9 were significantly associated with a shorter RFS, supporting the hypothesis that higher metabolic heterogeneity reflects a more aggressive tumor phenotype. Tumors exhibiting greater metabolic variability may contain hypoxic regions, necrotic areas, and clonal subpopulations with distinct metabolic profiles, all of which contribute to disease progression and poor response to therapy [19,20].

Although the International Prognostic Index (IPI) and its modified versions (revised IPI, NCCN-IPI, and others) remain widely used prognostic scoring systems in DLBCL, they have certain limitations. The IPI, despite its widespread acceptance, relies on a multiparametric structure in which each variable is assigned equal weight without subcategorization, which may reduce its predictive precision. Several efforts have been made over the years to revise and improve this scoring system [21,22,23]. In our cohort, the IPI score was not significantly associated with survival, possibly due to the limited sample size. By contrast, the HI-2, derived from PET/CT, demonstrated a significant association with survival and may serve as a complementary or alternative tool for risk stratification.

These findings have significant clinical implications. Identifying high-risk patients with DLBCL before initiating therapy is crucial for optimizing treatment strategies. Our study suggests that patients with high MTV and HI may benefit from intensified frontline therapies, such as dose-adjusted chemotherapy or early integration of targeted agents. Additionally, PET/CT-derived biomarkers can aid in monitoring the treatment response, enabling dynamic adjustments to therapy based on real-time metabolic changes. In our study, failure to achieve a complete or near-complete response on the first interim PET/CT analysis was associated with increased mortality. In our cohort, the rate of complete or near-complete response observed at interim analysis was 48%. In the meta-analysis by Adams et al., complete response rates ranged from 52% to 85% across studies. Although the response rate observed in our study appears to be lower than those reported in the literature, this may be attributed to the limited sample size. Adams et al. reviewed and conducted a meta-analysis of nine studies on the prognostic value of interim PET/CT in patients with DLBCL treated with R-CHOP. The predictive value was uniformly suboptimal across the studies [24]. This approach aligns with the principles of precision medicine, in which treatment strategies are tailored to an individual patient’s tumor biology.

Kwon et al. indicated that HI2 is an independent predictor of OS in oral cavity cancer. Patients with higher HI2 levels showed a worse prognosis than those with lower HI2 levels [14]. Kim et al. found that the survival of patients with heterogeneous tumors (HI2) was poorer than that of patients with relatively homogeneous tumors. Zhou et al., Esfahani et al., and Ceriani et al. estimated that TLG was the only independent predictor rather than MTV and SUVmax. In contrast, four other studies that analyzed only baseline MTV concluded that MTV was an independent predictor of PFS [25,26,27,28,29,30]. Xie et al. analyzed baseline MTV and TLG and concluded that both were independent predictors [31].

We investigated the prognostic value of MTV and HI in patients with DLBCL. Our findings largely agree with data in existing literature and support the main conclusions of previous comprehensive descriptions. Strong evidence exists in the literature regarding the use of MTV as a prognostic marker. As MTV indirectly reflects tumor aggressiveness and tumor burden, numerous studies examining the relationship between MTV and survival exist. Xie et al. (2016) emphasized that the MTV measured using FDG-PET/CT is an independent prognostic factor in DLBCL. Patients with a higher MTV had shorter survival times [31]. In addition, the determinant role of MTV in disease progression and survival was demonstrated in studies by Sasanelli et al. (2014) and Casasnovas et al. (2012) [29,30]. Additionally, studies on the prognostic value of the HI obtained using FDG-PET/CT have increased in recent years. Our study did not find a significant relationship between MTV and OS or RFS. However, we found a strong relationship between survival and HI2, which was calculated based on MTV. An HI2 value > 87.9 was associated with a shorter RFS. An oral cavity cancer study conducted by Kwon et al. (2014) showed that high HI2 values were associated with poor prognosis, and this finding may also be valid for DLBCL [14].

Similarly, Kim et al. (2015) revealed that heterogeneity factor values better reflect tumor biology in patients with breast cancer and play an essential role in survival prediction [11]. These results indicate that increased heterogeneity is associated with tumor aggressiveness and exhibits similar prognostic features in different cancer types. In our study, the biochemical and inflammatory marker LDH was associated with relapse rates. Several studies have reported that high LDH levels are associated with poor prognosis. For example, in a study by Zhou et al. (2016), LDH effectively predicted survival together with FDG-PET parameters, such as TLG and MTV [25]. Similarly, Chihara et al. (2011) showed that high SUVmax shortens survival in patients with DLBCL [32]. In our study, patients with a higher SUVmax had lower survival rates, which is consistent with the findings of previous studies. In a study by Liu X et al. on patients with colorectal cancer, a significant relationship was observed between HI2 expression and survival [33]. Liu G et al. reported a significant relationship between HI2 and survival in patients with gastric cancer [13]. In a study conducted by Chung et al. in patients with uterine cervical cancer, a significant relationship was observed between FDG-based intratumoral heterogeneity and survival [34]. In a study by Lee et al., a significant relationship was observed between FDG-based intratumoral heterogeneity and survival in patients with epithelial ovarian cancer [35].

In our study, stage, Ki-67, and LDH did not appear to be significantly associated with overall survival. However, it is well established that advanced-stage disease (particularly stage III–IV) is associated with poor prognosis in DLBCL, as clearly demonstrated in the original IPI, R-IPI, and NCCN-IPI studies [21,22,23]. The discrepancy between our findings and the existing literature may be attributed to the limited sample size. Ki-67 is also widely recognized as an indicator of aggressive behavior in lymphomas and other oncologic malignancies. Huixia et al. reported a strong association between Ki-67 and survival outcomes [36]. In contrast, the impact of Ki-67 on survival appeared limited in our analysis. This inconsistency may be due to the relatively low cut-off value (70%) used to stratify Ki-67 levels in our study.

In conclusion, the findings of our study support the data in the literature that parameters, such as MTV and HI obtained from PET/CT, are strong prognostic indicators of DLBCL. Our view that FDG-PET/CT can reflect not only the tumor’s metabolic activity but also its heterogeneous structure is consistent with similar studies conducted in different types of cancer [13,33,34,37,38]. However, larger-scale, prospective studies are needed to determine the standard threshold values for MTV and HI and to validate them in different patient groups. In addition, our study’s Kaplan–Meier and ROC curve analyses corroborated the findings of previous studies. In our study, a low MTV (SUVmax^liver^) was associated with decreased survival, consistent with previous findings.

This study has a few limitations. First, because this was a retrospective, single-center study with a relatively small sample size (n = 70), the generalizability of our findings is limited. Larger, multicenter prospective studies are needed to validate our results and establish more robust prognostic thresholds for MTV and HI in DLBCL. Second, variations in PET/CT acquisition protocols, image reconstruction techniques, and segmentation methods may introduce inconsistencies in MTV and HI measurements. Although efforts have been made to standardize imaging analyses, inter-institutional differences remain a challenge in the broader application of PET-derived biomarkers. Future studies should focus on the development of uniform and reproducible MTV and HI quantification methods to improve their clinical applicability. Third, although we identified significant associations between metabolic parameters and survival outcomes, potential confounding factors, such as subsequent treatment regimens, genetic and molecular characteristics of DLBCL subtypes, and individual patient responses to therapy, were not fully accounted for. These factors may influence prognosis independently of MTV and HI, warranting further investigation in future studies. Finally, owing to the retrospective nature of the study, longitudinal changes in MTV and HI over time were not assessed. Evaluating how these biomarkers evolve during treatment and their predictive value in dynamic response assessments could provide additional clinical insights. Future research should explore the roles of MTV and HI in guiding adaptive treatment strategies, particularly in response-adaptive therapy models. Despite these limitations, our study provides valuable evidence supporting the prognostic significance of the MTV and HI in DLBCL and highlights the need for further research to optimize their clinical utility. In contrast, some studies have focused on the PET/CT-based parameters of tumors detectable by PET/CT to distinguish poor prognosis and have achieved promising results in determining prognosis [32,39,40,41].

Despite these promising results, several challenges remain in translating MTV and HI into routine clinical use. One major limitation is the lack of standardized measurement protocols for these parameters across institutions. Variability in PET/CT acquisition settings, segmentation techniques, and thresholding methods can lead to inconsistencies in MTV and HI calculations. Additionally, prospective multicenter studies with larger sample sizes are needed to validate our findings and determine the optimal cut-off values for risk stratification.

## 5. Conclusions

In this study, we comprehensively evaluated the prognostic significance of PET/CT-based metabolic parameters, particularly MTV, HI, and SUVmax^liver^, as well as inflammation-related biomarkers, such as LDH and dNLR, in patients with DLBCL. Our findings demonstrate that both SUVmax^liver^ and HI2 are independent and powerful predictors of OS and RFS.

An SUVmax^liver^ value < 23 was significantly associated with a reduced OS, suggesting that lower liver metabolic activity may reflect aggressive tumor biology and resistance to therapy. Similarly, HI2 values > 62.3 were also associated with an increased mortality, while HI2 values > 87.9 were significantly predictive of relapse. Higher HI2 values indicate greater intratumoral metabolic heterogeneity, likely due to hypoxic regions, necrosis, and clonal diversity, which are associated with poor prognosis and limited therapeutic responses.

Furthermore, the inflammation-based marker LDH showed prognostic relevance. Patients with LDH levels > 302 IU/L had a significantly shorter RFS, underscoring the role of LDH as an indirect marker of tumor burden and proliferation.

ROC curve analyses confirmed the diagnostic utility of SUVmax^liver^, HI2, and LDH in predicting both mortality and relapse, with statistically significant AUC values. Multivariate logistic regression further established SUVmax^liver^ ≤ 22 and HI2 > 62.3 as independent predictors of mortality, while HI2 > 87.9 emerged as an independent predictor of relapse. These results underscore the potential of PET/CT-derived and inflammatory biomarkers to enhance risk stratification and guide clinical decision-making in DLBCL. Additionally, prospective multicenter studies with larger sample sizes are needed to validate our findings and determine the optimal cut-off values for risk stratification.

Our study supports the idea that volumetric and heterogeneity-based imaging biomarkers can offer prognostic insights beyond traditional clinical indices, such as the IPI, disease staging, and baseline LDH levels. Integrating MTV and HI into clinical workflows may help identify high-risk patients earlier, allowing for tailored treatment approaches and improved outcomes. Additionally, if HI-2 and SUVmax^liver^ are incorporated into routine clinical practice, they could be easily calculated with the aid of a simple software tool.

In conclusion, PET/CT-derived metabolic biomarkers, particularly SUVmax^liver^ and HI2, alongside inflammatory markers, such as LDH, are promising tools for risk prediction in DLBCL. Incorporating these parameters into prognostic models could facilitate the early identification of high-risk patients, support response-adaptive treatment strategies, and promote the implementation of precision medicine for managing lymphoma.

## Figures and Tables

**Table 1:** Demographic data of patients.

Characteristic	N	%
Age > 60	38	54.3
Age ≤ 60	32	45.7
Male	41	58.6
Female	29	41.4
Stage	N	%
Stage 1	14	20.0
Stage 2	9	12.9
Stage 3	12	17.1
Stage 4	35	50.0
Advanced Stage (3 and 4)	N	%
Yes	47	67.1
No	23	32.9
>1 Extranodal Site	N	%
Yes	23	32.9
No	47	67.1
Regimen	N	%
R-EPOCH	12	17.1
R-CHOP	58	82.9
ECOG PS	N	%
Score 1	33	47.1
Score 2	20	28.6
Score 3	17	24.3
ECOG ≥2	N	%
Yes	37	52.9
No	33	47.1
Treatment Response	N	%
Response Present	68	97.1
No Response	2	2.9
Response Type	N	%
Complete or Near Complete	34	48.6
Others	36	51.4
Mortality	N	%
Yes	21	30.0
No	49	70.0
Relapse	N	%
Yes	29	41.4
No	41	58.6
Elevated LDH	N	%
Yes	42	60.0
No	28	40.0
IPI Group	N	%
Low/Low-Intermediate	45	64.3
High-Intermediate/High	25	35.7
Bone Marrow Infiltration	N	%
Yes	5	7.1
No	65	92.9
Ki67	N	%
≥70%	37	63.8
<70%	21	36.2
Bulky Disease	N	%
Yes	15	21.4
No	55	78.6

## Data Availability

The data supporting the findings of this study can be obtained upon request from the corresponding author.

## Abbreviations

TMV	Tumor Metabolic Volume
DLBCL	Diffuse Large B-Cell Lymphoma
SUV	Standardized Uptake Value
HI	Heterogenity Index
RFS	Relapse-Free Survival
OS	Overall Survival
IPI	International Prognostic Index
NLR	Neutrophil-to-Lymphocyte Ratio
ROI	Region of İnterest
ECOG	Eastern Cooperative Oncology Group
SPSS	Statistical Package for the Social Sciences
ROC	Receiver Operating Characteristic
AUC	Area Under the Curve
HR	Hazard Ratio
